# Orthotist involvement in early gait rehabilitation following stroke: a cross sectional survey of orthotists in the United Kingdom

**DOI:** 10.1097/PXR.0000000000000365

**Published:** 2024-06-14

**Authors:** Miriam Golding-Day, Joshua Young, Paul Charlton, Brian Houston, Shirley Thomas, Marion Walker

**Affiliations:** 1 Centre of Rehabilitation and Ageing Research, The University of Nottingham, Nottingham, UK; 2 John Florence Orthotic Centre, Sussex, UK; 3 School of Health & Life Sciences, Teesside University, Middlesbrough, UK; 4 Peacocks Medical Group, Newcastle, UK

**Keywords:** Orthotist, Gait, Lower limb orthotics, Stroke rehabilitation, Multi-disciplinary team working

## Abstract

**Background:**

The role of the orthotist in early gait rehabilitation following stroke within the United Kingdom (UK) is currently poorly understood.

**Objectives:**

The objective of this study was to capture current opinion and practice of orthotists on their role in early stroke gait rehabilitation in the UK.

**Methods:**

An anonymous web-based cross-sectional survey was developed and distributed to Health and Care Professions Council (HCPC) registered orthotists via the mailing list of the British Association of Prosthetists and Orthotists (BAPO) professional body in the UK. Survey items were multiple choice, Likert scale or open-ended questions to explore the experiences and opinions of orthotists in early post-stroke gait rehabilitation.

**Results:**

Responses were received from 56 participants. Orthotists reported having regular contact with stroke patients within their usual clinical caseload. Multidisciplinary care was not typical with (46%) respondents ‘rarely’ seeing stroke patients in joint assessment with another clinician. Confidence in managing lower limb gait difficulties was high, with 89% of orthotists feeling able to recommend a lower limb orthotic treatment. Ninety-eight percent (n=55) of respondents agreed that orthotic assessment should be an essential element of gait re-education after stroke, however, orthotists reported limited involvement within early stroke rehabilitation.

**Conclusion:**

UK Orthotists surveyed in this study report that orthotic treatment is an important aspect of early gait rehabilitation following stroke. Respondents report barriers to multidisciplinary working which may affect outcomes in this patient group.

## Introduction

In the United Kingdom (UK), stroke is the leading cause of adult disability, frequently causing impairments in gait and mobility. (1) For those affected by stroke, the recovery of walking ability has been identified as a key goal in regaining their independence and progressing their rehabilitation. (1) Rehabilitation of gait following stroke may include various types of physical therapy, lower limb casting, medical management such as botulinum toxin, and the use of orthoses. An ankle-foot orthosis (AFO) is the most commonly used orthosis in stroke rehabilitation to assist with gait rehabilitation within an inpatient rehabilitation context, AFO use is between 22-39%. (2–4) AFOs are specifically designed to aid in the recovery of standing and walking ability and have been found to improve gait kinematics and kinetics, walking speed, energy expenditure and reduce risk of falls in people affected by stroke. (5,6) In Scotland, NHS Quality Improvement formed a working party of orthotists, physiotherapists, stroke nurses, bioengineers, staff from NHS Quality Improvement and patient representatives to create a Best Practice Statement (BPS) for use of AFOs following stroke. (7) This statement explored the function of AFOs in aiding rehabilitation in gait, finding AFOs were a key component of promoting early gait rehabilitation for some patients following stroke.

Within the UK the orthotist is the specialist clinician trained to assess for and prescribe orthoses such as AFOs and give biomechanical advice and input to aid with early gait rehabilitation following stroke. The Scottish BPS highlights the importance of the orthotists’ expertise in assessment, provision and on-going review of orthotic prescription as an important element of acute and longer-term post-stroke rehabilitation.

A study conducted in 2018-19 investigated the views of occupational therapists, orthotists and physiotherapists on orthotic intervention following stroke. (8) The study findings supported the BPS in identifying the important role orthotists are perceived to have within the stroke rehabilitation MDT. However, orthotists themselves are not currently identified as part of the core stroke rehabilitation multidisciplinary team (MDT) in the UK’s national clinical guidelines, which recommend ‘timely access to’ orthotic intervention as part of the stroke rehabilitation programme, but the mechanisms to facilitate this are unspecified. (9) Similarly, the English National Institute for Health and Care Excellence stroke rehabilitation guideline does not include orthotists in the core stroke rehabilitation MDT. (10)

One factor affecting the involvement of orthotists in early stroke care may be the availability of orthotic services; a study comparing UK orthotic services reported varied access to services. (11) In a stroke context, the study by Golding-Day and colleagues also reported varied access to orthotic services in different rehabilitation centres. (8)

The involvement of orthotists in early gait rehabilitation following stroke across the UK remains unclear, and the degree to which they influence recovery outcomes for patients both through the prescription and provision of orthotic devices and by working within the stroke rehabilitation MDT needs to be explored. This study aims to:

1. Investigate the opinions of orthotists on their role in early gait rehabilitation following stroke in the UK.
2. Capture current practices and the perspective of orthotists on their confidence to recommend orthotic treatment within the stroke rehabilitation pathway.
3. Revisit the findings from the national survey conducted in 2018-19 with a clearer focus on orthotists’ perspectives of their involvement and current practice specifically.

## Methods

### Study Design

A cross-sectional web-based survey was conducted containing 20 questions formatted to be answered via multiple choice, Likert scales or free text with additional space provided for other comments. The self-administered survey was designed to investigate practice and perceptions of certified prosthetist/orthotists within the UK concerning their involvement in early gait rehabilitation following stroke. Survey questions were divided into three sections:

Part 1 contained 5 questions recording participant characteristics;

Part II contained 7 questions capturing participants current clinical orthotic interactions within stroke care;

Part III contained 8 questions measuring participants confidence and perceptions of their role in gait rehabilitation following stroke.

The survey questions in their base form are available from the authors upon request. The survey was open to responses for 6-months, between April – September 2022. Prior to circulation, questions were edited and refined following a pilot and review process with eight clinical colleagues.

### Participants

The target population were certified prosthetist/orthotists either practicing or recently retired (within 5 years) based the UK. The British Association of Prosthetists and Orthotists (BAPO) is the national professional body of prosthetist/orthotists and holds the largest database of registered professionals within the country. A link to the survey was circulated to registered practicing members (n=434) and recently retired members (n=17) of BAPO via their mailing list. The survey was also advertised via a QR code on BAPO social media platforms (Facebook and Twitter). The inclusion criteria were:

- Qualified prosthetist/orthotist
- Post-qualification experience of working within adult stroke rehabilitation
- Currently working in the UK, or recently retired (within 5 years).

Eligibility was self-determined, and the survey was built to confirm eligibility before participants were able to advance through the questions.

### Ethical considerations

The survey was developed in collaboration with clinical and academic colleagues based within the UK, Sweden and Denmark. It was designed to take approximately 10 minutes to complete and to adhere to strict confidentiality of participants and their responses. The study was approved by Teesside University Health Research Ethics sub-Committee (2021-Feb-1723) and was piloted and approved for distribution by the BAPO Research Committee.

### Data analysis

Data was exported to Stata SE Version 13. Descriptive statistics were used to summarise the quantitative data, these can be found in tables 1-3.

This study was conducted in parallel with Danish colleagues who were investigating orthotists’ perspectives on their role in early post-stroke gait rehabilitation in Denmark, and the findings of this are published elsewhere. (12)

## Results

Responses were received from 56 participants, a response rate of 12%. Participant demographics are presented in Table 1. As some of the participants worked in multiple services, questions 5-12 were repeated for one (n=1) or two (n=1) additional services, giving a total of 59 responses.

### Participant professional demographics

Table 1 shows all respondents were HCPC registered orthotists in clinical practice. Most were qualified to degree level (73%), with 16% educated to Master’s level. Participants varied in experience, with 70% having six or more years’ experience and 45% having more than ten. Most orthotists described their primary employer as a private company providing NHS clinics (64%) or the NHS (29%). The clinical settings in which participants mostly work were mainly hospital – outpatients (57%) or hospital – ward (19%).

### Frequency, setting and timing of orthotic stroke care

Table 2 shows how most orthotists saw stroke patients regularly, from ‘Several times a month’ to ‘About once a day’ by 83% of respondents, with ‘Several times a week’ being the most frequent response (35%). Stroke patients were seen more than once per day by 5% and once per month or less by 11%. Stroke patients were usually seen as out-patients in a general orthotic clinic (43%) or as in-patients on a stroke or general ward within the acute hospital site (26%). Care was sometimes provided in more dedicated clinics: in MDT clinics by 21% or as out-patients in a dedicated neurological clinic by 11%. When defining MDT as ‘at least 2 different professions treating the patient in the same clinic, including orthotist’, it was most frequently reported that respondents saw stroke patients in this context ‘rarely’ (46%) or ‘some of the time’ (25%). The stage at which participants usually see patients following stroke varied, with 33% at 1-6 weeks, 22% at 7-12 weeks, 25% at 4-12 months and 19% >12 months.

### Orthotist involvement in early gait rehabilitation

Table 3 shows that orthotists most frequently reported that they are involved in early gait rehabilitation decisions being made ‘only when a specific referral for orthotic assessment is made by another MDT member’ (68%), with 27% reporting ‘I am not involved at all’ and 5% reporting ‘I am routinely involved without a specific referral for orthotic assessment being made’. When involved with early gait rehabilitation for stroke patients, assessment and detailed prescription decisions were most frequently made with the MDT (49%) or independently by the orthotist (38%). Similarly, use of the orthosis was usually determined with the MDT (52%) or independently by the orthotist (34%).

### Orthotist confidence in early gait rehabilitation

Table 4 shows how confidence in lower limb management was high amongst respondents, with 89% agreeing or strongly agreeing that they were confident in recommending a lower limb orthotic treatment plan within early stroke rehabilitation, and 83% agreeing or strongly agreeing they were confident advising on use of the orthosis. Confidence in other types of orthotic intervention was lower, with 73% agreeing or strongly agreeing that they were confident in recommending a comprehensive orthotic treatment plan to the MDT. Confidence in recommending therapy and physical activity was low; in response to the statement ‘I am confident advising therapy and activity to progress potential recovery (as a lead member of the therapy team)’ the most frequent responses were ‘neutral’ (38%) or ‘disagree’ (30%).

### Orthotist involvement in early gait rehabilitation

Table 5 shows how dissatisfaction with involvement in early stroke rehabilitation was frequently reported with 61% of respondents disagreeing or strongly disagreeing with the statement ‘I am satisfied with the frequency of involvement I have with early stroke rehabilitation patients.’ A further 23% described being neutral and only 16% were satisfied with their involvement. The majority (89%) of orthotists reported that they felt they should be routinely involved without a specific referral for orthotic assessment being made, by joining MDT meetings or routine assessment clinics. Almost all respondents (98%) agreed that orthotic assessment should be an essential element of gait re-education as part of recovery in stroke.

## Discussion

The purpose of this cross-sectional web-based survey was to explore the involvement of orthotists in early gait rehabilitation following stroke across the UK, and to capture the orthotists perspective on their ability to influence recovery outcomes via orthotic assessment and provision of orthotic devices within the stroke rehabilitation pathway. The results indicate that orthotists are involved with the early gait rehabilitation of stroke patients but that this involvement is predominantly in the outpatient setting. This corresponds to their orthotic assessment and treatment occurring later in the rehabilitation pathway once patients have been discharged from the acute setting. Whilst MDT working is considered the gold standard for complex rehabilitation interventions, (9) 46% of respondents reported that they rarely treated patients within an MDT setting.

This raises questions as to the method of specialist orthotic intervention during early gait rehabilitation and what can be done to better facilitate the orthotist role within the established stroke rehabilitation MDT. Current guidelines for gait specific and wider stroke rehabilitation do not include the orthotist within the named rehabilitation MDT, (9) leaving the means and method of their involvement ambiguous and open to localised interpretation. The findings from this survey support the need for clearer guidelines for orthotist involvement in gait rehabilitation following stroke, with orthotists intervening earlier on the pathway and within MDTs.

When orthotists are involved with early gait rehabilitation after stroke it is apparent that this is usually determined by members of the wider MDT, most commonly facilitated by a specific referral request. This method for facilitating specialist orthotic access is therefore dependent on given persons and their personal determinants of gait rehabilitation intervention, which may act in some cases as a barrier to the orthotist being able to provide care. This is due in part to a lack of clear best practice guidance with regards to orthotist involvement after stroke with current practice heavily dependent on localised relationships and procedures within a team. Only 5% of respondents reported routinely assessing patients for orthotic need during early gait rehabilitation, without a specific referral being made. This will also affect the timing of any orthotic intervention if it is undertaken at a later stage within the patient rehabilitation journey, and delays in orthotic intervention have been highlighted as potentially leading to poorer outcomes for stroke survivors, impacting on their rehabilitation more widely. (13)

As an opportunity to revisit the previous MDT survey conducted in 2019, (8) which focused on use of orthotic devices after stroke, the findings from this survey show confidence in recommending lower limb management amongst orthotists, indicating the strength of their specialist training in biomechanical principals and relevance to gait rehabilitation after stroke. Lower levels of confidence were reported in other areas of gait therapy and activities following stroke which is reflective of the orthotists reduced participation with the wider rehabilitation MDT, which creates a barrier to the development of further clinical skills and knowledge. Low numbers of orthotists practicing nationally as well as time and service pressures likely contribute towards this model of reduced MDT working.

Dissatisfaction with orthotist involvement in early stroke rehabilitation was highlighted with most orthotists reporting that they felt they should be routinely involved with the population of stroke patients who require gait rehabilitation without a referral being made. Due to their specialist biomechanical training, orthotists are best placed to determine if an orthotic device would be beneficial for a patient in early gait training but their ability to make such determinations are too heavily dependent on the views of the wider stroke rehabilitation team as the referring party. Almost all respondents (n= 55, 98%) agreed that orthotic assessment should be an essential element of gait re-education as part of recovery in stroke, making it an area where changes to current practice should be focused. The present study in based on the UK context, however the parallel study conducted in Denmark found similar results, suggesting that some of the issues considered may be relevant internationally. (12) Danish orthotists reported involvement late in the rehabilitation process, and also reported the belief that they should be involved at an earlier stage as part of gait rehabilitation.

### Study strengths and limitations

This is the first survey of UK orthotists on their involvement with early gait rehabilitation following stroke, addressing a gap in the literature. Additionally, there is very little data on this topic internationally, with the study by Jakobsen being the only other example of which the authors are aware. (12) The inclusion of orthotists in the research team is beneficial in ensuring relevant issues are addressed, when considering the clinical practice of orthotists. Limitations include the response rate of 12%, which is relatively low and leads to a high level of non-response bias. Respondents were also likely self-selective as those that are interested in stroke rehabilitation and this will be reflected in their responses. Moreover, the survey was circulated to members of BAPO and whilst they are the professional body for orthotists practicing in the UK, membership is not a requirement and so there will be orthotists who will have been missed in the participant approach and so their views will not be reflected in the results.

Another limitation of this study was the close ended nature of the questions. The survey was developed by the investigators to capture current practice and opinions of the orthotists on their role. We pilot-tested the survey and revised the questions and their format based on feedback that the survey needed to be swift to complete, potentially between busy clinical caseloads, and to encourage a greater response rate. The data collected from the open-ended questions was not included in the scope of the current paper.

## Conclusion

UK Orthotists responding to this survey report that orthotic treatment is an important aspect of early gait rehabilitation following stroke, but current practice appears to be varied and based on localised practice. Variation in practice may be related to discontinuity between existing guidance. (7,9,10) Clear clinical pathways for orthotic involvement to aid gait training following stroke would assist in providing a blueprint for services to be structured in a way which enables greater MDT interaction which could improve outcomes for stroke survivors with gait difficulties. Clinical guidelines detailing specific expected orthotist involvement pathways after stroke, informed by evidence-based research and consensus, would be beneficial to reduce variation in care.

UK Orthotists reported dissatisfaction in current pathways and the belief that earlier involvement in the rehabilitation process would benefit patients, which also reflects the views of orthotists surveyed elsewhere. (12) There is currently a paucity of research and professional consensus evidence in this area which needs to be addressed before recommendations can be actioned. Specific considerations for complex conditions such as stroke within orthotic service organisation and funding would be useful to appropriately address the needs of this patient group and foster closer working relationships with the wider stroke rehabilitation MDT, which may improve clinical outcomes for stroke survivors.

## Figures and Tables

**Table 1:** Participant professional demographics

Professional role	n	(%0
Orthotist	56	(100)

**Highest degree or level of education**		
Diploma	6	(11)
Degree	41	(73)
Masters	9	(16)
PhD	0	(0)

**Years qualified**		
0-2 years	4	(7)
3-5 years	13	(23)
6-10 years	14	(25)
More than 10 years	25	(45)

**Primary employer**		
National Health Service (NHS)	17	(30)
Private company providing NHS clinics	37	(66)
Private company providing private clinics	2	(4)
		

**Table 2:** Frequency, setting and timing of orthotic stroke care

Clinical settings (all that apply)	n = 86	%
Hospital setting – Ward	16	19
Hospital setting – Outpatients	49	57
Clinical in building not on hospital site	13	15
Community team seeing people at their usual place of residence (home, care home, etc.)	3	3
Private clinic	4	5
Other	1	1

**Frequency of treating stroke patients**	**n=59**	**%**
More than one a day	3	5
About once a day	12	20
Several times a week	21	36
About once a week	10	17
Several times a month	6	10
About once a month	5	8
Less frequently than once a month	2	4

**Clinical setting stroke patients seen in (all that apply)**	**n=117**	**%**
I see stroke patients as out-patients in a general orthotic clinic	50	43
I see stroke patients as out-patients in a dedicated neurological clinic	13	11
I see stroke patients as in-patients on a stroke or general ward within the acute hospital site	30	25
I see stroke patients within a dedicated in-patient stroke service in a Multi-Disciplinary Team (MDT) setting	10	9
I see stroke patients within a dedicated out-patient stroke service in a MDT setting	8	7
I see stroke patients within a dedicated community stroke service in a MDT setting	6	5

**Regularity of MDT assessment of stroke patients (*defined as: at least 2 different professions treating the patient in the same clinic, including orthotist*)**	**n=59**	**%**
Never	10	17
Rarely	27	46
Some of the time	15	25
Often	5	9
All of the time	2	3

**Length of time after stroke when patient assessed by orthotist**	**n=58**	**%**
1-2 weeks	4	7
3-6 weeks	15	26
7-12 weeks post stroke	13	22
4-6 months post stroke	13	22
7-12 months post stroke	2	4
>12 months post stroke	11	19

**Table 3:** Orthotist involvement in early gait rehabilitation

Involvement in early gait rehabilitation decisions for stroke patients	n=59	%
I am not involved at all	16	27
I am involved but only when a specific referral for orthotic assessment is made by another MDT member	40	68
I am routinely involved without a specific referral for orthotic assessment being made (e.g. I join the MDT meetings or routine assessment clinic)	3	5

**Decision making for early gait rehabilitation orthotic assessment and prescription (all that apply)**	**n=65**	**%**
Other members of the MDT or the referrer formulates the orthotic prescription	8	12
I work with the MDT to formulate the orthotic prescription	32	49
I formulate the orthotic prescription independently	25	39

**Orthotic use determined by **	**n=65**	**%**
Other members of the MDT or the referrer will determine and advise the patient how the orthosis should be best used.	9	14
I work with the MDT to determine and advise the patient how the orthosis should be best used.	34	52
I independently determine and advise the patient how the orthosis should be best used.	22	34

**Table 4:** Orthotist confidence and perception of role

I am Confident in recommending a comprehensive orthotic treatment plan to the stroke rehabilitation MDT	n=56	%
Strongly Disagree	1	2
Disagree	7	12.5
Neutral	7	12.5
Agree	23	41
Strongly Agree	18	32

**Orthotic assessment should be an essential element of gait re-education as part of recovery**	**n=55**	**%**
Strongly Disagree	1	2
Disagree	0	0
Neutral	0	0
Agree	15	27
Strongly Agree	39	71

**I am confident in recommending a lower limb orthotic treatment plan within early stroke rehabilitation**	**n=56**	**%**
Strongly Disagree	2	4
Disagree	1	2
Neutral	3	5
Agree	27	48
Strongly Agree	23	41

**I am confident advising on use of the orthosis within an MDT including duration of use both daily and longer term**	**n= 55**	**%**
Strongly disagree	1	2
Disagree	2	4
Neutral	6	11
Agree	20	36
Strongly agree	26	47

**I am confident advising therapy and activity to progress potential recovery (as a lead member of the therapy team)**	**n= 56**	**%**
Strongly disagree	4	7
Disagree	17	30
Neutral	21	38
Agree	9	16
Strongly Agree	5	9

**I am satisfied with the frequency of involvement I have with early stroke rehabilitation patients**	**n= 56**	**%**
Strongly disagree	13	23
Disagree	21	38
Neutral	13	23
Agree	5	9
Strongly agree	4	7

**Table 5:** Orthotist involvement in early gait rehabilitation

Opinion of involvement of orthotist in early gait rehabilitation decisions (*early rehabilitation identified as within six weeks of onset*)	n = 56	%
I don’t think Orthotists should be involved at all	0	0
I think Orthotists should be routinely involved without a specific referral for orthotic assessment being made (e.g. join the MDT meetings or routine assessment clinic)	50	89
I think Orthotists should be involved but only when a specific referral for orthotic assessment is made by another MDT member	6	11

**I am satisfied with the frequency of involvement I have with early stroke rehabilitation patients**	**n= 56**	**%**
Strongly disagree	13	23
Disagree	21	38
Neutral	13	23
Agree	5	9
Strongly agree	4	7
